# Triangulating evidence on the role of perceived versus objective experiences of childhood adversity in psychopathology

**DOI:** 10.1111/jcv2.12010

**Published:** 2021-04-28

**Authors:** Jessie R. Baldwin, Michelle Degli Esposti

**Affiliations:** ^1^ Department of Clinical, Educational and Health Psychology Division of Psychology and Language Sciences University College London London UK; ^2^ Social, Genetic and Developmental Psychiatry Centre Institute of Psychiatry, Psychology and Neuroscience King’s College London London UK; ^3^ Department of Social Policy and Intervention University of Oxford Oxford UK

## INTRODUCTION

Childhood adversities, such as maltreatment, bullying, and socioeconomic deprivation, are well‐established risk factors for psychopathology. Recent evidence suggests that it is the perceived, rather than objective (i.e., actual) experience of childhood adversity which is associated with psychopathology (Danese & Widom, 2020). However, it is unclear whether perceptions of childhood adversity *cause* psychopathology, as this cannot be tested ethically or feasibly with randomised controlled trials. Triangulation can instead be used to improve causal inference in observational research, by integrating evidence across multiple approaches with different sources of bias.

**Key points**

- Childhood adversity is associated with psychopathology, but it is unclear whether risk of psychopathology is driven by the objective or perceived experience of adversity.
- To strengthen causal inference on the role of perceived versus objective experiences of childhood adversity in psychopathology, it is important to triangulate findings across multiple approaches.
- We review evidence addressing this question from three complementary approaches with different sources of bias—measurement comparisons, within‐family comparisons and cross‐context comparisons.
- Triangulating evidence from measurement, within‐family and cross‐context comparisons provides considerable (though not complete) support for a role of perceived experience of child adversity in psychopathology, independent of objective experience.
- Future research is required to address sources of inconsistencies in current evidence and reach a definitive conclusion.

In this perspective, we describe three different approaches—measurement comparisons, within‐family comparisons, and cross‐context comparisons—that can be used to test the role of perceived versus objective experiences of childhood adversity in psychopathology. We review current evidence from each approach, before triangulating findings to strengthen causal inference. A summary of each approach and its potential sources of bias is in Table 1.

**TABLE 1:** Sources of bias in different approaches to studying the role of perceived versus objective experience in psychopathology

Approach, studies	Description of approach	Potential sources of bias
Measurement comparison (Bouman et al., 2012; Danese & Widom 2020; Goldman‐Mellor et al., 2016; Graham & Juvon, 1998; Gromann et al., 2013)	Compares the relative contribution of objective versus subjective measures (of the same experience) in risk of psychopathology. This can be performed via multivariate regression or stratification across groups categorised according to objective versus subjective evidence of exposure.	Confounding by unmeasured risk factors for psychopathology which differ between those with objective versus subjective evidence of experiences. Misclassification bias (e.g., low sensitivity of objective measures) could result in underestimates of the association between objective measures of childhood adversity and psychopathology. However, this was not likely to account for findings in Danese and Widom (2020) because (i) results were consistent across different types of maltreatment with differences in sensitivity of objective measures, and (ii) reclassification of participants was unlikely to affect the results. Also, misclassification bias is unlikely to affect findings on peer victimisation (e.g., Boumann et al., 2012), because objective measures are based on peer nominations (i.e., anonymous reports from all children in a class). Treatment effects in those with objective measures (e.g., court records of maltreatment or recognised bullying) could lower risk of subsequent psychopathology. However, if this was the case, lower risk of psychopathology would also be expected in those with both objective and subjective evidence (which was not found in Danese & Widom, 2020; Graham & Juvonen, 1998; Gromann et al., 2013). Similarly, the association between subjective measures and psychopathology would be attenuated when controlling for the objective measure (which was not the case in Goldman‐Mellor et al., 2016). Recall bias (if subjective reports of exposure are assessed concurrently to psychopathology). Reverse causality (if earlier psychopathology causes biased perceptions of environments).
Within‐family comparison (Newbury et al., 2017; Rivenbark et al., 2020)	Tests whether twins or siblings who differ in their perceptions of environments that are shared between them (e.g. family or neighbourhood exposures) differ in psychopathology risk. Controls for confounding by genetic influences (100% MZ twins; 50% DZ twins/siblings) and the shared family environment.	Family/neighbourhood environments may differ between twins or siblings (e.g., if one family member is exposed to more childhood adversity than the other). If this is the case, reported “perceived” experiences may partly capture objective experience. Confounding by individual risk factors for psychopathology which differ between family members (unlikely to be the case in Rivenbark et al. (2020) which controlled for prior mental health, child intelligence and negative affect). If familial factors (e.g., heritable traits) strongly influence perceptions of environments, there will be little within‐pair variance left to detect an association between measures of perceived childhood adversity with psychopathology. Recall bias (if subjective reports of exposure are assessed concurrently to psychopathology). Reverse causality (if earlier psychopathology causes biased perceptions of environments).
Cross‐context comparison (Gershoff et al., 2010, 2012; Lansford et al., 2005, 2014)	Tests whether the association between experiences and psychopathology is stronger in contexts in which the experience is perceived as harmful. This is achieved by comparing results between two (or more) populations in different contexts, or from the same population over different time periods, in which the perceived normativeness of an experience (e.g., physical discipline) varies.	Confounding by a third variable (e.g., parental psychopathology), which differs across contexts. For example, if parental psychopathology is associated with the use of physical discipline in contexts where physical discipline is non‐normative (but not where it is normative), parental psychopathology might confound associations between physical discipline and psychopathology in non‐normative contexts only. As a result, the association between physical discipline and child psychopathology would be inflated in non‐normative contexts, and the effect of perceived experience would be over‐estimated. Measurement of the exposure and/or outcome are not equivalent across the populations/contexts being compared. However, all studies assessed the same disciplinary behaviours and used the same mental health measures, adapted for different languages (Gershoff, Grogan‐Kaylor, et al., 2010; Gershoff, Lansford, et al., 2012; Lansford, Chang, et al., 2005; Lansford, Sharma, et al., 2014). If perceived norms (e.g., acceptability of physical discipline) are not directly measured in the study, perception of the experience as harmful may be incorrectly inferred for the different populations/contexts. However, this was not an issue in two studies (Gershoff, Grogan‐Kaylor, et al., 2010; Lansford, Chang, et al., 2005), as the perceived normativeness of the experience was assessed by asking children and parents about the frequency in which other parents use discipline strategies.

Abbreviations: MZ, monozygotic; DZ, dizygotic.

## MEASUREMENT COMPARISONS

Measurement comparisons test the relative associations between subjective and objective assessments of childhood adversity (e.g., self‐reports vs. official records) with psychopathology. This is achieved by including subjective and objective measures as predictors in multivariate regression analyses, or by stratifying by measurement type and comparing risk of psychopathology. Naturally, measurement comparisons can only be made in cohorts which have both subjective and objective measures of childhood adversity, which are relatively rare as objective measures (e.g., official child protection records) can be challenging to obtain (Danese & Widom, 2020).

Nevertheless, a handful of studies have capitalised on such cohorts to test the relative associations between perceived and objective experiences of various childhood adversities (maltreatment, bullying, and neighbourhood violence) with psychopathology. Regarding maltreatment, Danese and Widom (2020) found that children with court‐documented abuse and neglect had minimal risk of psychopathology in adulthood, if they did not self‐report maltreatment as adults. In contrast, those who self‐reported maltreatment had elevated risk of psychopathology irrespective of court records. Regarding bullying, three studies found that children who self‐reported peer victimisation had higher levels of psychopathology, independent of more objective measures (peer nominations; Bouman et al., 2012; Graham & Juvonen, 1998; Gromann et al., 2013). Conversely, peer nominations of victimisation were not associated with psychopathology in the absence of self‐report. Regarding neighbourhood violence, adolescents who perceived their neighbourhood to be unsafe reported elevated psychological distress, independent of more objective measures (neighbourhood violent crime records; Goldman‐Mellor et al., 2016). However, neighbourhood crime records alone were not associated with psychological distress. Collectively, these findings suggest that perceived childhood adversity is associated with psychopathology independent of objective experience, but not vice versa.

As described in detail in Table 1, measurement comparisons can involve various sources of bias. For example, individuals with pre‐existing psychiatric vulnerabilities (e.g., genetic risk) may be more likely to perceive environments negatively and develop psychopathology. Additionally, 'objective' measures (e.g., official records of maltreatment) can have low sensitivity, and thus may under‐estimate the association between objective experience and psychopathology. Therefore, in addition to accounting for these biases through statistical analyses, it is important to contrast findings from measurement comparisons to those from other approaches with unrelated biases.

## WITHIN‐FAMILY COMPARISONS

Within‐family comparisons examine whether perceptions of family or neighbourhood environments are associated with psychopathology, beyond objective experiences. This involves testing whether twin/sibling differences in perceived environments that are shared (e.g., family/neighbourhood conditions) are associated with twin/sibling differences in psychopathology. Because twins and siblings grow up in the same household, this approach aims to control for objective family and neighbourhood conditions by design. Furthermore, because twins and siblings share genetic material and other familial environments, within‐family comparisons also control for these familial confounders.

Within‐twin comparisons have been used to examine the unique role of perceived neighbourhood adversity and family social status in psychopathology. Newbury et al. (2017) found that twins who perceived higher levels of neighbourhood disorder than their co‐twin had greater risk of psychotic experiences in adolescence. Additionally, Rivenbark et al. (2020) found that adolescents who perceived their family’s social status as lower than their co‐twin had poorer mental health, including more symptoms of depression, anxiety, and conduct problems. This association was independent of individual‐level risk factors including prior mental health and childhood intelligence.

Within‐family comparisons are not immune from bias (see Table 1). In particular, the association between perceived experiences and psychopathology could be over‐estimated if twins/siblings objectively experience differences in family or neighbourhood environments. This is unlikely to affect evidence focusing on adversities that equally affect children in a family (e.g., low socioeconomic status), but is more likely for adversities that can differ between children (e.g., maltreatment or parenting, as shown in twin studies; Fisher et al., 2015). Additionally, results from within‐family comparisons could be confounded by individual factors (i.e., risk factors for psychopathology which differ between relatives), if these are not measured and controlled for (as in Rivenbark et al., 2020). Notably, these sources of bias are not present in the third approach: cross‐context comparisons.

## CROSS‐CONTEXT COMPARISONS

Cross‐context comparisons exploit variation in societal norms, to test whether experiences are associated with psychopathology only in contexts in which they are perceived as harmful. One example of an adverse childhood experience that is perceived differently across cultural and historical contexts is physical discipline. For example, in some countries, physical discipline is considered to be normative (e.g., Malaysia; Kumaraswamy & Othman, 2011), while in others, it is an illegal violation of a child’s rights (e.g., Sweden). Therefore, if physical discipline is associated with psychopathology in Sweden but not in Malaysia, it would suggest that the perceived harm of the experience (rather than the objective experience) drives poor mental health. A cross‐historical approach could also be used to compare associations between physical discipline and psychopathology in a single country over time, as smacking children has become less normative (Degli Esposti et al., 2019).

Four studies have used a cross‐cultural approach to examine the role of perceived physical discipline in psychopathology, while a cross‐historical approach has not yet been used. Lansford et al. (2005) compared the association between physical discipline and child behaviour problems across 6 countries (China, India, Italy, Kenya, the Philippines and Thailand) which differed in the normative use of physical discipline (as reported by parents and children). The study found that countries with the lowest reported normative use of physical discipline (Thailand and China) had the strongest association between discipline and child behaviour problems, suggesting that perceived experience was associated with psychopathology. A later study (Lansford et al., 2014) also found that corporal punishment was more strongly associated with child anxiety in cultural groups with less traditionally authoritarian parenting (e.g., Latin Americans in the United States vs. the Philippines). In contrast, however, Gershoff et al. (2010) found that associations between physical disciplinary methods and child behaviour problems did not differ across countries with varying reported norms towards parental discipline. Similarly, Gershoff et al. (2012) found that associations between spanking and child externalising behaviour did not vary across ethnic/racial groups (White, Black, Hispanic and Asian American) with previously observed differences in normative use of spanking. This evidence therefore provides mixed support for an association between perceived (over objective) experiences of physical discipline in psychopathology.

Cross‐context comparisons might be affected by three main sources of bias (Table 1). First, if confounding differs across contexts, any differences in the associations between physical discipline and psychopathology between contexts might reflect differential confounding, rather than the perceived harm of the experience. Second, if measures of physical discipline and/or mental health differ between the contexts, differences in associations between contexts might reflect measurement discrepancies. However, all studies assessed the same disciplinary behaviours and used consistent psychopathology measures across contexts. Third, if norms regarding physical discipline are not directly measured in the sample (which was the case in Gershoff et al. [2012] and Lansford et al. [2014], but not in the other studies), perceived harm of the experience could be incorrectly inferred across different contexts.

## TRIANGULATION OF EVIDENCE

Triangulating evidence from measurement, within‐family and cross‐context comparisons provides considerable (though not complete) support for a role of perceived experience of child adversity in psychopathology, independent of objective experience. Findings from measurement and within‐family comparisons unanimously suggest that this is the case (Bouman et al., 2012; Danese & Widom, 2020; Graham & Juvonen, 1998; Gromann et al., 2013; Newbury et al., 2017; Rivenbark et al., 2020), indicating that findings are unlikely to reflect biases specific to either approach. Evidence from cross‐context comparisons is less consistent, with two studies suggesting that perceived experience is associated with psychopathology (Lansford et al., 2005, 2014) and two studies suggesting no independent role of perceived experience (Gershoff et al., 2010, 2012). These discrepant cross‐context findings may reflect limitations of specific studies (e.g., incorrect assumptions about cultural norms, or low power in small samples to test for interactions by context; Gershoff et al., 2010). It is also possible that discrepant findings between the two (negative) cross‐context comparisons with measurement and within‐family comparisons reflect confounding by individual‐specific vulnerabilities in measurement/within‐family comparisons (which is unlikely to affect cross‐context comparisons). However, this explanation is less plausible given that findings from two other cross‐context comparisons were consistent with measurement and within‐family comparisons (Lansford et al., 2005, 2014). Therefore, triangulation of evidence to‐date broadly suggests that perceived experience of childhood adversity is associated with risk of psychopathology, independent of objective experience. However, because the number of available studies is modest, and some discrepancies exist, future research is needed to reach a definitive conclusion.

## IMPLICATIONS FOR RESEARCH AND TREATMENT

We suggest three directions for future research. First, studies that address potential sources of discrepant findings in current evidence (e.g., confounding by individual factors, or limitations of specific cross‐context comparisons) will be valuable to resolve current ambiguities. In addition to the approaches described here, alternative approaches could be used. For example, experimental methods (e.g., using virtual reality or therapeutic techniques to manipulate objective vs subjective experience) could be used to examine the impact of perception on mental health outcomes under tightly controlled conditions. Comparisons could also be made across historical contexts with differing norms regarding physical discipline (similarly to cross‐cultural comparisons). Second, because different types of adverse childhood experiences have been studied across approaches, future studies should examine the subjective versus objective effect of same adversity type across different approaches, for more focused triangulation. Third, studies which examine multiple types of childhood adversity using the same approach could indicate whether the role of perceived versus objective experience differs according to the type of adverse childhood experience.

Understanding the relative contribution of perceived versus objective experience of childhood adversity in psychopathology can provide new directions for mental health intervention. For example, if psychopathology develops due to the perceived experience, therapeutic approaches which target subjective appraisal could minimise the impact of childhood adversity on psychopathology. If psychopathology develops primarily due to the objective experience, then primary prevention of childhood adversity could help to reduce the prevalence of mental health problems. Of course, primary prevention of childhood adversity is essential regardless of the mental health consequences, but understanding the contribution of subjective versus objective experiences can inform strategies for psychiatric intervention.

## AUTHOR CONTRIBUTIONS

*Conceptualization, data curation, investigation, methodology, project administration, writing‐original draft, writing‐review & editing*: Jessie R. Baldwin. *Conceptualization, data curation, investigation, methodology, project administration, writing‐original draft, writing‐review and editing*: Michelle Degli Esposti.

## Data Availability

Not applicable as this editorial perspective reviews current evidence rather than conducting data analysis.
